# Exploration of *CYP21A2* and *CYP17A1* polymorphisms and preeclampsia risk among Chinese Han population: a large-scale case-control study based on 5021 subjects

**DOI:** 10.1186/s40246-020-00286-0

**Published:** 2020-09-25

**Authors:** Bo Hou, Xuewen Jia, Ziwen Deng, Xin Liu, Huitang Liu, Haichu Yu, Shiguo Liu

**Affiliations:** 1 Department of Cardiology, The Affiliated Hospital of Qingdao University, Qingdao, China; 2 Emergency Department, Shengli Oilfield Central Hospital, Dongying, China; 3 Medical Genetic Department, The Affiliated Hospital of Qingdao University, Qingdao, China; 4 Prenatal Diagnosis Center, The Affiliated Hospital of Qingdao University, Qingdao, China; 5 Department of Blood Transfusion, The First Affiliated Hospital of Zhengzhou University, Zhengzhou, China; 6 Department of Laboratory Medicine, Zhongnan Hospital of Wuhan University, Wuhan, China

**Keywords:** Preeclampsia, GWAS, *CYP17A1*, *CYP21A2*, Susceptibility

## Abstract

**Background:**

Several genome-wide association studies have identified single-nucleotide polymorphisms (SNPs), such as rs4409766, rs1004467, and rs3824755 in *CYP17A1* and rs2021783 in *CYP21A2*, as new hypertension susceptibility genetic variants in the Chinese population. This study aimed to look into the relationship between preeclampsia (PE) and these SNPs in Chinese Han women.

**Methods:**

Overall, 5021 unrelated pregnant women were recruited, including 2002 patients with PE and 3019 normal healthy controls. The real-time PCR (TaqMan) method was applied to genotype these four polymorphisms.

**Results:**

A statistically obvious difference in the allelic frequencies was observed in CYP21A2 rs2021783 between cases and controls (*χ*^2^ = 7.201, *Pc* = 0.028 by allele), and the T allele was associated with the occurrence and development of PE (OR = 1.151, 95% CI 1.039–1.275). We also found a significant association between *rs2021783 and the development of* early-onset PE (*Pc* = 0.008 by genotype, *Pc* = 0.004 by allele). For rs1004467 and rs3824755, the distribution of allelic frequencies differed markedly between mild PE and control groups (*χ*^2^ = 6.843, *Pc* = 0.036; *χ*^2^ = 6.869, *Pc* = 0.036), and patients with the TT genotype of rs1004467 were less easy to develop mild PE than were those carrying the CT or CC genotype (*χ*^2^ = 7.002, *Pc* = 0.032, OR = 1.306, 95% CI 1.071–1.593). The GG genotype of rs3824755 appeared to a protective effect on the occurrence of mild PE (OR = 0.766, 95% CI 0.629–0.934).

**Conclusions:**

CYP21A2 rs2021783 appears to be closely related to PE susceptibility, and CYP17A1 rs1004467 and rs3824755 seem to be closely associated with mild PE in Han women.

## Background

Preeclampsia (PE) is a special complication of pregnancy that is characterized by de novo proteinuria and hypertension after 20 gestational weeks. It affects 2 to 8% of all pregnant woman worldwide and is the leading cause of maternal and fetal morbidity and mortality [1, 2], especially in developing countries. Furthermore, women with PE are more likely to develop cardiovascular disease (CVD), hypertension, and chronic kidney disease in later life [3, 4]. As a special type of hypertension, PE is a complex multifactor disease, which is the result of the interaction between genetic factors and environmental factors. Therefore, factors that affect blood pressure may be one of the primary mechanisms underlying PE. Although many environmental risk factors are associated with the formation of hypertension, including higher sodium intake, smoking, excessive alcohol consumption, obesity, activation of the sympathetic nervous system, and endothelial dysfunction [5], genetic factors also play an important role in the maintenance of hypertension [6]. Furthermore, multiple studies have shown that many candidate hypertension genes [7–9], such as *STOX1*, *IL-1*, *IL6*, and *COMT*, were also shown to be linked to PE [10–13].

Recent genome-wide association studies (GWAS) identified many genetic variants or chromosomal regions that are associated with hypertension, such as *ATP2B1*, *CYP17A1*, *CYP1A2*, *SH2B3*, *CACNB2*, *TGFB2*, *MTHFR*, *CYP11B2*, and *ULK4* [14–17]. Furthermore, *ATP2B1*, *MTHFR*, and *CYP11B2* have been validated to have associations with the risk of PE in different ethnic groups [18–20]. Two large GWAS meta-analyses suggested that rs1004467 (*P* = 1.28 × 10^−10^) and rs3824755 (*P* = 1.21 × 10^−6^) in *CYP17A1* were significantly associated with the risk of hypertension [14, 16]. Subsequently, Lu et al. found that rs4409766 had the strongest correlation with *CYP17A1* (*P* = 7.33 × 10^−13^) and *CYP21A2* (*P* = 3.53 × 10^−11^) in a large-scale hypertension GWAS, of which 80,962 subjects were Chinese Han nationality [21].

*CYP17A1* encodes the cytochrome P450 protein, which has 17-alpha-hydroxylase and 17,20-lyase activities and is one of the key enzymes in the synthesis of human steroid hormones. It had been investigated to play a role in multiple complex diseases, such as cardiovascular disease [22], hypertension [14], and fetal growth restriction [23]. *CYP21A2* encodes 21-hydroxylase enzyme, and mutations in this gene may lead to congenital adrenal hyperplasia, which is characterized by hypertension, hypokalemia, and sexual infantilism [24]. Because they are candidate genes for blood pressure and play crucial roles in steroid hormone biosynthesis, thus having a further effect on hypertension, we designed and conducted this study to explore the relationship between genetic variations in *CYP17A1* (rs4409766, rs1004467, and rs3824755) and *CYP21A2* (rs2021783) and the sensitivity of Chinese Han women to PE.

## Material and methods

### Subjects

A total of 5021 subjects, including 2002 PE patients (mean age 30.09 ± 5.65 years) and 3019 controls (mean age 30.31 ± 4.01 years), were recruited from the Affiliated Hospital of Qingdao University, Linyi People’s Hospital, Liaocheng People’s Hospital, Yantai Yuhuangding Hospital, Heze Municipal Hospital, Binzhou Medical University Hospital, and the Maternal and Child Health Care of Zaozhuang between January 2012 and May 2016. All women had singleton pregnancies and were of Chinese Han ethnicity. The inclusion criteria for cases were established according to ACOG (American College of Obstetricians and Gynecologists, 2013), defined as hypertension (systolic blood pressure ≥ 140 mmHg or diastolic blood pressure ≥ 90 mmHg on two or more occasions at least 4 h apart) with proteinuria (0.3 g/24 h, 0.3 mg/dl, or 1+ by dipstick, without renal disease or infection) after 20 gestational weeks [25]. Exclusion criteria for both groups included chronic hypertension, a history of renal and endocrinal diseases, multifetal pregnancy, and autoimmune disorder. The control groups included normal healthy pregnant women without any pregnancy disorder or fetal disorders. All PE subjects were divided into the mild PE group (*n* = 454) and severe PE group (*n* = 1548). Severe PE was diagnosed if any following symptoms appeared in the case subjects: blood pressure ≥ 160/110 mmHg or progressive renal insufficiency (proteinuria ≥ 5 g/24 h, or cr > 1.1 mg/dl), new-onset visual or cerebral disturbances, impaired liver functions, and pulmonary edema [25]. Additionally, we divided the cases into early-onset PE (*n* = 937, before 34 weeks of gestation) and late-onset PE (*n* = 1065, after 34 weeks of gestation).

The questionnaire provided clinical data, including age, history of pregnancy and childbirth, blood pressure, gestational age, clinically relevant symptoms, and some laboratory examinations. All participants were provided with an explanation of the research and then gave written informed consent. Our study was approved by the ethics committee of the Affiliated Hospital of Qingdao University.

### Genotyping

Qiagen DNA extraction kit (Qiagen, Hilden, Germany) was applied to extract genomic DNA from 2 ml peripheral venous anticoagulant blood, and then stored at – 80 °C. SNP genotyping was determined by the TaqMan allele recognition real-time PCR. The probes and primers were designed and synthesized by Applied Biosystems by Life Technologies (ABI, NY, USA). The sequence of primers was provided in https://www.mysequenom.com/Tools. All polymerase chain reaction (PCR) amplification systems (thermocycler, C1000TM) were conducted in a total of 25 μl, containing DNase-free water, 11.25 μl of DNA, and 12.5 μl of 2× PCR Master Mix and 1.25 μl of 20× SNP Genotyping Assay. The cycling protocol was included in an initial denaturation at 95 °C for 3 min, and then carried out 45 cycles of 95 °C for 15 s and 60 °C for 1 min. The fluorescent signals of VIC/FAM-labeled probes were detected in each cycle, and different genotypes were detected applying Bio-Rad CFX manager 3.0 software. We also selected 100 samples of each genetic variant to verify the genotype by Sanger sequencing.

### Statistical analysis

All data were analyzed applying the software package SPSS 22.0 (SPSS Inc., Chicago, IL, USA). Clinical data and demographic were performed using Student’s *t* test or the chi-square test and were described by the mean ± standard error (SE) or percentage. A *P* value < 0.05 was considered statistically significant. Each control group polymorphism was tested for the Hardy-Weinberg equilibrium using the goodness-of-fit *χ*^2^ test. The distributions of genotypic and allelic frequencies between the case and control groups were analyzed by Pearson’s *χ*^2^ test. Ninety-five percent confidence intervals (CIs) and odds ratios (ORs) were applied to express the risk between the groups, and *P* < 0.05 was considered statistically significant; however, for four SNPs, a level of *P* < 0.0125 or *Pc* < 0.05 was considered significant when using Bonferroni’s correction. The genotype-phenotype was analyzed by the one-way analysis of variance. The power analysis was calculated using the program Power and Sample Size Calculations (PS, version 3.1.2).

## Results

### Demographic and clinical characteristics

In Table 1, the demographic and clinical features of the two groups were shown. There were no obvious differences in the age, age of menarche, or number of abortions between the control group and the case group (*P* > 0.05). However, compared with normal controls, PE patients were more likely to have earlier gestational weeks at admission and gestational weeks at delivery, lower fetal birth weight, lower cholesterol, higher blood pressure, higher triglyceride, higher creatinine, elevated white blood cells, and higher blood urea nitrogen (*P* < 0.001).

**Table 1:** Demographic and clinical characteristics of the case and control groups

	Case	Control	*t*	*P*
Age (years)	30.09 ± 5.65	30.31 ± 4.01	− 1.478	0.140
Age of menarche (years)	13.98 ± 1.19	14.02 ± 1.20	− 0.984	0.325
Number of abortion	0.62 ± 0.95	0.62 ± 0.86	− 0.102	0.919
Gestational age at admission (weeks)	35.56 ± 3.48	39.16 ± 1.35	− 43.19	0.000*
Gestational age at delivery (weeks)	36.21 ± 3.09	39.36 ± 1.22	− 41.25	0.000*
Fetal birth weight (g)	2600 ± 934	3387 ± 673	− 30.66	0.000*
Systolic blood pressure (mmHg)	160.05 ± 18.51	115.88 ± 10.56	95.73	0.000*
Diastolic blood pressure (mmHg)	104.12 ± 13.63	73.47 ± 7.32	91.24	0.000*
White blood cell (WBC)	9.61 ± 2.84	8.96 ± 2.41	8.26	0.000*
Triglyceride	3.88 ± 1.83	2.94 ± 1.25	11.85	0.000*
Cholesterol (CHO)	3.89 ± 1.87	6.11 ± 1.23	− 27.88	0.000*
Blood urea nitrogen (BUN)	4.67 ± 1.98	3.18 ± 0.98	28.80	0.000*
Creatinine (CREA)	65.26 ± 27.60	51.46 ± 19.66	17.45	0.000*

*Significant difference vs. controls

### Genetic analysis

The genotypic distributions of the following SNPs in the control groups were consistent with the Hardy-Weinberg equilibrium (for rs4409766, *χ*^2^ = 0.091, *P* = 0.763; for rs1004467, *χ*^2^ = 1.203, *P* = 0.272; for rs3824755, *χ*^2^ = 0.206, *P* = 0.886; for rs2021783, *χ*^2^ = 0.040, *P* = 0.841).

The genotypic and allelic frequencies of *CYP17A1* (rs4409766, rs1004467, rs3824755) and *CYP21A2* (rs2021783) polymorphisms are summarized in Table 2.

**Table 2:** Genotypic and allelic distributions in the case and control groups

		Cases, *N* = 2002	Controls, *N* = 3019	*χ*^2^	*P*	*Pc*	OR (95% CI)
rs4409766							
Genotypes	TT	1018 (50.85)	1509 (49.98)	2.518	0.284	**–**	
	CT	826 (41.25)	1233 (40.84)				
	CC	158 (7.90)	277 (9.18)				
Alleles	T	2862 (71.47)	4251 (70.40)	1.345	0.246	0.984	1.054 (0.965~1.151)
	C	1142 (28.43)	1787 (29.60)				
rs1004467							
Genotypes	CC	259 (12.94)	419 (13.90)	1.424	0.491	**–**	
	CT	920 (45.95)	1401 (46.40)				
	TT	823 (41.11)	1199 (39.70)				
Alleles	C	1438 (35.91)	2239 (37.08)	1.414	0.234	0.936	0.951 (0.875~1.033)
	T	2566 (64.09)	3799 (62.92)				
rs3824755							
Genotypes	GG	856 (42.76)	1232 (40.81)	2.392	0.302	**–**	
	GC	908 (45.35)	1396 (46.24)				
	CC	238 (11.89)	391 (12.95)				
Alleles	G	2620 (65.43)	3860 (63.93)	2.386	0.122	0.488	0.936 (0.861~1.018)
	C	1384 (44.57)	2178 (36.07)				
rs2021783							
Genotypes	CC	1362 (68.03)	1936 (64.13)	8.151	0.017	0.068	
	CT	567 (28.32)	961 (31.83)				
	TT	73 (3.65)	122 (4.04)				
Alleles	C	3291 (82.19)	4833 (80.04)	7.201	**0.007**^a^	**0.028**^a^	1.151 (1.039~1.275)
	T	713 (17.81)	1205 (19.96)				

*Pc P* value corrected by Bonferroni’s method

^a^Significant difference vs. controls

There was an obvious difference in the allelic frequencies of *CYP21A2* rs2021783 between the PE patients and control group (*χ*^2^ = 7.201, *P* = 0.007, *Pc* = 0.028). The T allele was related to the occurrence of PE (OR = 1.151, 95% CI 1.039–1.275). In the genotype (CC vs. CT vs. TT, *P* = 0.017; however, after Bonferroni’s correction, *Pc* = 0.068), the difference between the groups showed no statistical significance. In contrast, there were no obvious differences in the genotypic and allelic frequencies of rs4409766, rs1004467, and rs3824755 in *CYP17A1* between the groups (for rs4409766, *χ*^2^ = 2.518, *P* = 0.284 by genotype, and *χ*^2^ = 1.345, *P* = 0.246 by allele; for rs1004467, *χ*^2^ = 1.424, *P* = 0.491 by genotype, and *χ*^2^ = 1.414, *P* = 0.234 by allele; and for rs3824755, *χ*^2^ = 2.392, *P* = 0.302 by genotype, and *χ*^2^ = 2.386, *P* = 0.122 by allele).

For rs2021783 in *CYP21A2*, Table 3 displays that there was no difference between the patients with mild/severe PE and control groups (*Pc* > 0.05). However, for rs1004467, the distribution of allelic frequencies differed significantly between the mild PE and control groups (*χ*^2^ = 6.843, *P* = 0.009, *Pc* = 0.036). The allele T may thus be a risk factor for mild PE (OR = 1.219, 95% CI 1.051–1.413), and the TT genotype was associated with the risk of developing mild PE compared to those subjects who had a CT or CC genotype (*χ*^2^ = 7.002, *P* = 0.008, *Pc* = 0.032, OR = 1.306, 95% CI 1.071–1.593). Similarly, the allelic frequencies of rs3824755 differed significantly between the mild PE group and the control group (*χ*^2^ = 6.869, *P* = 0.009, *Pc* = 0.036). Further, the GG genotype appeared to be a protective effect on the occurrence of mild PE (OR = 0.766, 95% CI 0.629–0.934). For rs4409766, there was no difference in the genotypic and allelic frequencies between the mild/severe PE and control groups (*P* > 0.05).

**Table 3:** Genotype and allele distributions in the mild/severe PE and control groups

SNP		Control	Mild PE	*χ*^2^	*P* value	*Pc*	OR	95% CI	Severe PE	*χ*^2^	*P* value	*Pc*	OR	95% CI
	*N*	3019	454						1548					
rs4409766	TT	1509	252						766					
	CT	1233	170						656					
	CC	277	32	5.508	0.064	0.256			126	1.879	0.651			
	T	4251	674						2188					
	C	1787	234	5.598	0.018	0.072	1.211	1.033–1.419	908	0.071	0.791		1.013	0.921–1.114
rs1004467	TT	1199	210						613					
	CT	1401	192						728					
	CC	419	52	7.330	0.026	0.104			205	0.390	0.823			
	T	3799	612						1954					
	C	2239	296	6.843	**0.009**^**a**^	**0.036**^**a**^	1.219	1.051–1.413	1142	0.034	0.855		1.008	0.922–1.103
rs3824755	GG	1232	215						641					
	GC	1396	191						717					
	CC	391	48	7.330	0.026	0.104			190	0.460	0.794			
	G	3860	621						1999					
	C	2178	287	6.869	**0.009**^**a**^	**0.036**^**a**^	1.221	1.051–1.418	1097	0.363	0.547		0.973	0.888–1.065
rs2021783	TT	122	12						61					
	CT	961	132						435					
	CC	1936	310	4.005	0.135	0.540			1052	6.982	0.030	0.120		
	T	1205	156						557					
	C	4833	752	3.862	0.049	0.196	1.202	1.000–1.444	2539	5.081	0.024	0.096	1.137	1.017–1.270

*Pc P* value corrected by Bonferroni’s method

^a^Significant difference vs. controls

*We also* found *that rs2021783* was *related to the risk of* early-onset PE (*Pc* = 0.008 by genotype, *Pc* = 0.004 by allele). *No obvious differences* were *found in the genotypic and allelic frequencies of CYP17A1 (rs4409766, rs1004467, and rs3824755) between the control group and early and advanced PE patients (Table* *4**).*

**Table 4:** Genotype and allele distributions in the early-onset/late-onset PE and control groups

		Control	Early-onset PE	*χ*^2^	*P* value	*Pc*	OR	95% CI	Severe PE	*χ*^2^	*P* value	*Pc*	OR	95% CI
	*N*	3019	937						1065					
rs4409766	TT	1509	460						558					
	CT	1233	405						421					
	CC	277	72	2.896	0.235				86	2.195	0.334			
	T	4251	1325						1537					
	C	1787	549	0.062	0.803		1.015	0.905–1.137	593	2.226	0.136		1.087	0.974–1.213
rs1004467	TT	1199	372						451					
	CT	1401	442						478					
	CC	419	123	0.385	0.825				136	2.460	0.292			
	T	3799	1186						1380					
	C	2239	688	0.083	0.773		1.016	0.912–1.131	750	2.374	0.123		1.084	0.978–1.202
rs3824755	GG	1232	385						471					
	GC	1396	445						463					
	CC	391	107	1.587	0.452				131	3.789	0.150			
	G	3860	1215						1405					
	C	2178	659	0.511	0.475		0.961	0.863–1.071	725	2.843	0.092		1.093	0.986–1.213
rs2021783	TT	122	35						38					
	CT	961	242						325					
	CC	1936	660	12.986	**0.002**^**a**^	**0.008**^**a**^			702	1.272	0.529			
	T	1205	312						401					
	C	4833	1562	10.099	**0.001**^**a**^	**0.004**^**a**^	1.248	1.088–1.431	1729	1.274	0.259		1.138	0.948–1.219
	CT	961	242						325					
	CC+TT	2058	695	12.183	**0.000**^**a**^	**0.000**^**a**^	0.746	0.632–0.880	740	0.631	0.427		1.063	0.914–1.237
	CC	1936	660						702					
	CT+TT	1083	277	12.622	**0.000**^**a**^	**0.000**^**a**^	1.333	1.137–1.562	363	1.101	0.294		0.924	0.798–1.071

*Pc P* value corrected by Bonferroni’s method

^a^Significant difference

### Analysis of genotype-phenotype relationship

A laboratory-based examination of PE among several genotypes is presented in *Table* *5**. For* rs2021783*, patients carrying the TT* genotype had a lower menarche age (13.55 ± 1.31 vs. 14.01 ± 1.09, or vs. 14.00 ± 1.22; *P* = 0.027) than did those with the CT or CC genotype. Furthermore, the level of urea nitrogen was lower in patients with the CT genotype than in those carrying the CC genotype (4.49 ± 1.72 vs. 4.73 ± 2.00, *P* = 0.016). However, the gestational age, birth weight of children, number of abortions, systolic/diastolic blood pressure, triglyceride, total cholesterol, and creatinine showed no statistical differences among three groups (all, *P* > 0.05).

**Table 5:** Laboratory-based examination of PE among different genotypes

	(1) TT	(2) CT	(3) CC	(1) vs. (2) vs. (3)	(1) vs. (2)	(1) vs. (3)	(2) vs. (3)	(1) + (2) vs. (3)	(1) vs. (2) + (3)
				*P*	*P*	*P*	*P*	*P*	*P*
Age (years)	30.15 ± 6.13	29.99 ± 5.51	30.12 ± 5.50	0.894	0.817	0.962	0.644	0.679	0.917
Age of menarche (years)	13.55 ± 1.31	14.01 ± 1.09	14.00 ± 1.22	**0.012**^**a**^	**0.027**^**a**^	**0.027**^**a**^	0.999	0.458	**0.003**^**a**^
Number of abortion	0.617 ± 0.90	0.615 ± 0.95	0.619 ± 0.95	0.981	0.984	0.952	0.051	0.844	0.97
Gestational age at admission (weeks)	35.62 ± 3.07	35.64 ± 3.65	35.49 ± 3.44	0.700	0.963	0.768	0.411	0.399	0.845
Gestational age at delivery (weeks)	36.12 ± 2.89	36.17 ± 3.32	36.24 ± 3.00	0.893	0.372	0.353	0.169	0.653	0.791
Fetal birth weight (g)	2603 ± 975	2616 ± 952	2593 ± 926	0.895	0.910	0.931	0.638	0.647	0.979
Systolic blood pressure (mmHg)	160.28 ± 17.38	159.80 ± 17.84	159.41 ± 17.84	0.854	0.830	0.686	0.662	0.604	0.724
Diastolic blood pressure (mmHg)	103.78 ± 12.58	103.62 ± 13.11	104.37 ± 13.90	0.533	0.927	0.718	0.272	0.263	0.819
White blood cell (×10^9^/l)	9.56 ± 2.81	9.70 ± 2.99	9.72 ± 3.08	0.910	0.704	0.666	0.927	0.835	0.672
Triglyceride (mmol/l)	3.82 ± 1.87	3.88 ± 1.69	3.91 ± 1.95	0.929	0.822	0.738	0.818	0.756	0.759
Total cholesterol (mmol/l)	6.69 ± 1.70	6.71 ± 1.95	6.72 ± 1.92	0.980	0.960	0.899	0.862	0.844	0.916
Urea nitrogen (mmol/l)	4.54 ± 1.77	4.49 ± 1.72	4.73 ± 2.00	**0.048**^**a**^	0.832	0.435	**0.016**^**a**^	**0.009**^**a**^	0.624
Creatinine (μmol/l)	61.69 ± 21.18	64.29 ± 25.48	65.39 ± 28.94	0.466	0.473	0.290	0.446	0.314	0.330

^a^Significant difference

## Discussion

A normal pregnancy is a condition of increased extracellular fluid, plasma volume, renal blood flow, and glomerular filtration rate. In contrast, PE is characterized by hypertension, proteinuria, higher vascular resistance, and decreased intravascular volume, cardiac output, and uteroplacental flow [26]. Despite unremitting efforts that have been undertaken for several decades, the precise pathophysiology or pathogenesis of this human pregnancy disorder remains elusive. However, increasing evidence suggests that gene-environment interactions maybe play important roles in the pathogenesis of PE [6]. Because PE is a special type of hypertension, we hypothesize that the genetic factors that affect blood pressure may be the primary cause for the incidence of PE.

GWAS had been demonstrated to be an effective way for identifying genetic variants in many common traits and diseases [27]. The Framingham Heart Study enrolled 9400 participants across three generations in Framingham, and the Wellcome Trust Case Control Consortium, which examined 2000 cases and a shared set of 3000 controls of white Europeans in Great Britain, reported the first two GWAS of hypertension but did not observe genetic loci that attained genome-wide significance [28, 29]. Then, a study in 34,433 subjects of European ancestry found eight blood pressure loci, including rs11191548 in *CYP17A1*, rs17367504 in *MTHFR*, rs12946454 in *PLCD3*, rs1378942 in *CYP1A2*, rs16998073 in *FGF5*, rs653178 in *SH2B3*, rs1530440 in *c10orf107*, and rs16948048 in *ZNF652* [15]. Since then, more genetic loci, such as *ATP2B1*, *CYP17A1*, *CYP1A2*, *SH2B3*, *CACNB2*, *MTHFR*, *CYP11B2*, and *ULK4*, were identified in a large-scale GWAS associated with blood pressure [14, 15, 30, 31].

Some genetic variants in *CYP17A1* were associated with hypertension in different populations, such as rs11191548 which was found to be associated with both the She ethnic minority in China and the Chinese Han population [32, 33]. rs11191416 and rs6163 were also identified to have association with blood pressure in 4178 European ancestry participants [34]. Furthermore, rs1004467 in *CYP17A1* is a common intronic variant that is associated with hypertension; it has been reported as a significant locus in the genome-wide meta-analysis and was verified in a Chinese Han cohort [15]. A study of mean arterial pressure and pulse pressure among 26,600 East Asians followed by a repeat study of 28,783 participants suggested that CYP17A1 rs3824755 was associated with blood pressure (*P* = 1.2 × 10^−6^) [16]. The other two genetic loci of CYP21A2 rs2021783 and CYP17A1 rs4409766 were only found to be associated with blood pressure in a Chinese population [21], which indicated that these SNPs were genetic marker variants of hypertension in Chinese populations.

However, in addition to genetic factors, metabolic factors such as abnormal steroid metabolism play important roles in the occurrence of PE [35]. In normal pregnancy, the aldosterone levels are elevated to contribute to sodium retention and the water retention, whereas aldosterone is reduced in PE patients [35, 36]. Additionally, PE patients had increased serum progesterone or androgen levels and decreased estrogen levels [37].

*CYP17A1* and *CYP21A2* play important roles in the metabolic pathways of steroid hormones. *CYP17A1* encoded the cytochrome P450 proteins, which had 17-alpha-hydroxylase and 17,20-lyase activities, which was the key step in the biosynthesis of mineralocorticoids, glucocorticoids, and sex-steroid biosynthesis [38]. *CYP21A2* encoded 21-hydroxylase enzyme and converted 17-hydroxyprogesterone into 11-deoxycortisol or progesterone to deoxycorticosterone [24]. Aldosterone affected blood pressure and the blood volume in the body by regulating the salt retained by the kidneys. Therefore, the mutations in *CYP17A1* and *CYP21A2* may lead to a loss in enzyme activity and then affect the synthesis of steroid hormones.

In our study, we investigated the associations between rs4409766, rs1004467, and rs3824755 in *CYP17A1* and rs2021783 in *CYP21A2* and PE; several GWAS had identified these as the genetic variants associated with hypertension in China [16, 21]. To our knowledge, *this* is *the first study to study the relationship between patients with PE and normal pregnant women of CYP17A1 and CYP21A2 in Chinese Han women. In th*e present study, the C allele of rs2021783 indicated an increased risk in patients with PE compared to the control group. The CC genotype was more susceptible to experiencing severe PE or early-onset PE. The CT genotype had the effect of protecting women from severe PE or early-onset PE. We also found, for rs1004467, that the T allele was the risk allele for the occurrence of mild PE and that patients with the TT genotype had a 1.219-fold risk of developing the PE compared to patients with CT or CC genotype in Chinese Han women. For rs3824755, the frequency of the G allele was associated with a higher risk of mild PE patients, and the GG genotype was identified as a risk factor in mild PE. However, Lim et al. investigated the association between polymorphisms of -34T/C in the promoter in *CYP17A1* and PE in 164 cases and 182 normal pregnancies in Korea but did not obtain significant results [39]. Additionally, Coto et al. found no relationship between *CYP21A2* rs6471 polymorphism with PE in 250 cases and 250 controls from Asturias [40].

*Although our study* contained *a sufficient number of samples, including 2002* PE patients and 3019 age-matched normal controls*, and* had sufficient statistical power *for* each SNP (rs1004467, 0.221; rs4409766, 0.214; rs3824755, 0.338) *to draw conclusions*, *some* limitations should be noted. *All subjects* were *selected from Shandong Province of China, and* a single ethnic group may yield false positives due to unmeasured collinearity in the cohort. Thus, our findings should be expanded outside the Han ethnicity and be validated in different ethnicities and regions. *Although PE* was *generally considered* a complex multifactorial disease resulting from genetic and environment risk factors, including dietary and lifestyle factors, we could not consider all of these factors. Additionally, whether other SNPs in *CYP21A2* and *CYP17A1* participate in the development of PE needs to be studied further.

## Conclusion

CYP21A2 rs2021783 appears to be closely associated with PE susceptibility, and CYP17A1 rs1004467 and rs3824755 seem to be related to mild PE in Chinese Han women.

## Data Availability

The dataset supporting the conclusions of this article is included within the article.

## Abbreviations

SNPs: Single-nucleotide polymorphisms
PE: Preeclampsia
CVD: Cardiovascular disease
GWAS: Genome-wide association studies
AGEN: Asian Genetic Epidemiology Network
CHARGE: Cohorts for Heart and Aging Research in Genomic Epidemiology
PCR: Polymerase chain reaction
SE: Standard error
ORs: Odds ratios
CIs: Confidence intervals

## References

[1] Duley L (2009). The global impact of pre-eclampsia and eclampsia. Semin Perinatol.

[2] Umesawa M, Kobashi G (2017). Epidemiology of hypertensive disorders in pregnancy: prevalence, risk factors, predictors and prognosis. Hypertens Res.

[3] Ahmed R, Dunford J, Mehran R, Robson S, Kunadian V. Pre-eclampsia and future cardiovascular risk among women a review. J Am Coll Cardiol 2014;63(18):1815-22 doi: 10.1016/j.jacc.2014.02.529 [published Online First: Epub Date]. 10.1016/j.jacc.2014.02.529 24613324

[4] Amiri M, Ramezani Tehrani F, Rahmati M, Behboudi-Gandevani S, Azizi F (2019). Changes over-time in blood pressure of women with preeclampsia compared to those with normotensive pregnancies: a 15year population-based cohort study. Pregnancy Hypertens.

[5] Whelton PK, He J, Appel LJ (2002). Primary prevention of hypertension - clinical and public health advisory from the National High Blood Pressure Education Program. JAMA.

[6] Garcia EA, Newhouse S, Caulfield MJ, Munroe PB (2003). Genes and hypertension. Curr Pharm Design.

[7] Allison SJ (2016). Hypertension: IL-1 receptor-induced sodium reabsorption in hypertension. Nature reviews. Nephrology.

[8] Htun NC, Miyaki K, Song YX, Ikeda S, Shimbo T, Muramatsu M (2011). Association of the catechol-o-methyl transferase gene Val158Met polymorphism with blood pressure and prevalence of hypertension: interaction with dietary energy intake. Am J Hypertens.

[9] Singh S, McDonough CW, Gong Y, et al. Genome wide association study identifies the HMGCS2 locus to be associated with chlorthalidone induced glucose increase in hypertensive patients. J Am Heart Assoc. 2018;7(6). 10.1161/JAHA.117.007339 published Online First: Epub Date. 10.1161/JAHA.117.007339 PMC5907544 29523524

[10] Stonek F, Hafner E, Metzenbauer M, et al. Absence of an association of tumor necrosis factor (TNF)-alpha G308A1 interleukin-6 (IL-6) G174C and interleukin-10 (IL-10) G1082A polymorphism in women with preeclampsia. J Reprod Immunol 2008;77(1):85-90 doi: 10.1016/j.jri.2007.04.003 [published Online First: Epub Date]. 10.1016/j.jri.2007.04.003 17544514

[11] Mohajertehran F, Afshari JT, Rezaieyazdi Z, Ghomian N (2012). Association of single nucleotide polymorphisms in the human tumor necrosis factor-alpha and interleukin 1-beta genes in patients with pre-eclampsia. Iran J Allergy Asthm.

[12] Li J, Liu MC, Zong JB (2014). Genetic variations in IL1A and IL1RN are associated with the risk of preeclampsia in Chinese Han population. Sci Rep-Uk.

[13] Erlandsson L, Ducat A, Castille J (2019). Alpha-1 microglobulin as a potential therapeutic candidate for treatment of hypertension and oxidative stress in the STOX1 preeclampsia mouse model. Sci Rep.

[14] Levy D, Ehret GB, Rice K (2009). Genome-wide association study of blood pressure and hypertension. Nat Genet.

[15] Newton-Cheh C, Johnson T, Gateva V (2009). Genome-wide association study identifies eight loci associated with blood pressure. Nat Genet.

[16] Kelly TN, Takeuchi F, Tabara Y, et al. Genome-wide association study meta-analysis reveals transethnic replication of mean arterial and pulse pressure loci. Hypertension 2013;62(5):853-59 doi: 10.1161/Hypertensionaha.113.01148 [published Online First: Epub Date]. 10.1161/HYPERTENSIONAHA.113.01148 PMC3972802 24001895

[17] Evangelou E, Warren HR, Mosen-Ansorena D, et al. Genetic analysis of over 1 million people identifies 535 new loci associated with blood pressure traits. Nat Genet 2018;50(10):1412-25 doi: 10.1038/s41588-018-0205-x [published Online First: Epub Date]. 10.1038/s41588-018-0205-x PMC6284793 30224653

[18] Wan JP, Wang H, Li CZ (2014). The common single-nucleotide polymorphism rs2681472 is associated with early-onset preeclampsia in northern Han Chinese women. Reprod Sci.

[19] Li X, Luo YL, Zhang QH (2014). Methylenetetrahydrofolate reductase gene C677T, A1298C polymorphisms and pre-eclampsia risk: a meta-analysis. Mol Biol Rep.

[20] Bogacz A, Bartkowiak-Wieczorek J, Procyk D, et al. Analysis of the gene polymorphism of aldosterone synthase (CYP11B2) and atrial natriuretic peptide (ANP) in women with preeclampsia. Eur J Obstet Gyn R B 2016;197:11-15 doi: 10.1016/j.ejogrb.2015.11.012 [published Online First: Epub Date]. 10.1016/j.ejogrb.2015.11.012 26686590

[21] Lu X, Wang L, Lin X (2015). Genome-wide association study in Chinese identifies novel loci for blood pressure and hypertension. Hum Mol Genet.

[22] Consortium IKC (2011). Large-scale gene-centric analysis identifies novel variants for coronary artery disease. PLoS Genet.

[23] Sata F, Yamada H, Suzuki K, et al. Functional maternal catechol-O-methyltransferase polymorphism and fetal growth restriction. Pharmacogenet Genomics 2006;16(11):775-81 doi: 10.1097/01.fpc.0000230116.49452.c0 [published Online First: Epub Date]. 10.1097/01.fpc.0000230116.49452.c0 17047485

[24] Falhammar H, Nordenstrom A. Nonclassic congenital adrenal hyperplasia due to 21-hydroxylase deficiency: clinical presentation, diagnosis, treatment, and outcome. Endocrine 2015;50(1):32-50 doi: 10.1007/s12020-015-0656-0 [published Online First: Epub Date]. 10.1007/s12020-015-0656-0 26082286

[25] American College of O, Gynecologists, Task Force on Hypertension in P. Hypertension in pregnancy. Report of the American College of Obstetricians and Gynecologists’ Task Force on Hypertension in Pregnancy. Obstetrics and gynecology 2013;122(5):1122-31 doi: 10.1097/01.AOG.0000437382.03963.88 [published Online First: Epub Date]. 10.1097/01.AOG.0000437382.03963.88 24150027

[26] Verdonk K, Visser W, van den Meiracker AH, Danser AHJ (2014). The renin-angiotensin-aldosterone system in pre-eclampsia: the delicate balance between good and bad. Clin Sci.

[27] McCarthy MI, Abecasis GR, Cardon LR, et al. Genome-wide association studies for complex traits: consensus, uncertainty and challenges. Nat Rev Genet 2008;9(5):356-69 doi: 10.1038/nrg2344 [published Online First: Epub Date]. 10.1038/nrg2344 18398418

[28] Burton PR, Clayton DG, Cardon LR, et al. Genome-wide association study of 14,000 cases of seven common diseases and 3,000 shared controls. Nature 2007;447(7145):661-78 doi: 10.1038/nature05911 [published Online First: Epub Date]. 10.1038/nature05911 PMC2719288 17554300

[29] Levy D, Larson MG, Benjamin EJ, et al. Framingham heart study 100K project: genome-wide associations for blood pressure and arterial stiffness. BMC Med Genet. 2007;8. 10.1186/1471-2350-8-S1-S3 published Online First: Epub Date. 10.1186/1471-2350-8-S1-S3 PMC1995621 17903302

[30] Kato N, Takeuchi F, Tabara Y, et al. Meta-analysis of genome-wide association studies identifies common variants associated with blood pressure variation in east Asians. Nat Genet 2011;43(6):531-8 doi: 10.1038/ng.834 [published Online First: Epub Date]. 10.1038/ng.834 PMC3158568 21572416

[31] International Consortium for Blood Pressure Genome-Wide Association S, Ehret GB, Munroe PB, et al. Genetic variants in novel pathways influence blood pressure and cardiovascular disease risk. Nature 2011;478(7367):103-9 doi: 10.1038/nature10405 [published Online First: Epub Date]. 10.1038/nature10405 PMC3340926 21909115

[32] Liu C, Li H, Qi Q (2011). Common variants in or near FGF5, CYP17A1 and MTHFR genes are associated with blood pressure and hypertension in Chinese Hans. J Hypertens.

[33] Lin Y, Lai X, Chen B, et al. Genetic variations in CYP17A1, CACNB2 and PLEKHA7 are associated with blood pressure and/or hypertension in she ethnic minority of China. Atherosclerosis 2011;219(2):709-14 doi: 10.1016/j.atherosclerosis.2011.09.006 [published Online First: Epub Date]. 10.1016/j.atherosclerosis.2011.09.006 21963141

[34] Morrison AC, Bis JC, Hwang SJ (2014). Sequence analysis of six blood pressure candidate regions in 4,178 individuals: the cohorts for heart and aging research in genomic epidemiology (CHARGE) targeted sequencing study. PLoS One.

[35] Verdonk K, Saleh L, Lankhorst S (2015). Association studies suggest a key role for endothelin-1 in the pathogenesis of preeclampsia and the accompanying renin-angiotensin-aldosterone system suppression. Hypertension.

[36] Escher G, Mohaupt M. Role of aldosterone availability in preeclampsia. Mol Asp Med 2007;28(2):245-254 doi: 10.1016/j.mam.2007.03.002 [published Online First: Epub Date]. 10.1016/j.mam.2007.03.002 17512581

[37] Salas SP, Marshall G, Gutierrez BL, Rosso P (2006). Time course of maternal plasma volume and hormonal changes in women with preeclampsia or fetal growth restriction. Hypertension.

[38] Auchus RJ (2001). The genetics, pathophysiology, and management of human deficiencies of P450c17. Endocrinol Metab Clin N Am.

[39] Lim JH, Kim SY, Kim DJ, et al. Genetic polymorphism of catechol-O-methyltransferase and cytochrome P450c17alpha in preeclampsia. Pharmacogenet Genomics 2010;20(10):605-10 doi: 10.1097/FPC.0b013e32833df033 [published Online First: Epub Date]. 10.1097/FPC.0b013e32833df033 20729792

[40] Coto E, Tavira B, Marin R, et al. Functional polymorphisms in the CYP3A4, CYP3A5, and CYP21A2 genes in the risk for hypertension in pregnancy. Biochem Biophys Res Commun 2010;397(3):576-9 doi: 10.1016/j.bbrc.2010.06.003 [published Online First: Epub Date]. 10.1016/j.bbrc.2010.06.003 20617557

